# Supplementary material for: Structure of Vibrio Phage XM1, a Simple Contractile DNA Injection Machine
Source: Viruses. 2023 Jul 31;15(8):1673. doi: 10.3390/v15081673 (PMC10457771; doi:10.3390/v15081673)
Supplement: Supplementary file 1 [file viruses-15-01673-s001.zip › viruses-2493093-supplementary.pdf]

Structure of *Vibrio* phage XM1, a simple contractile DNA injection machine

SUPPLEMENTARY INFORMATION

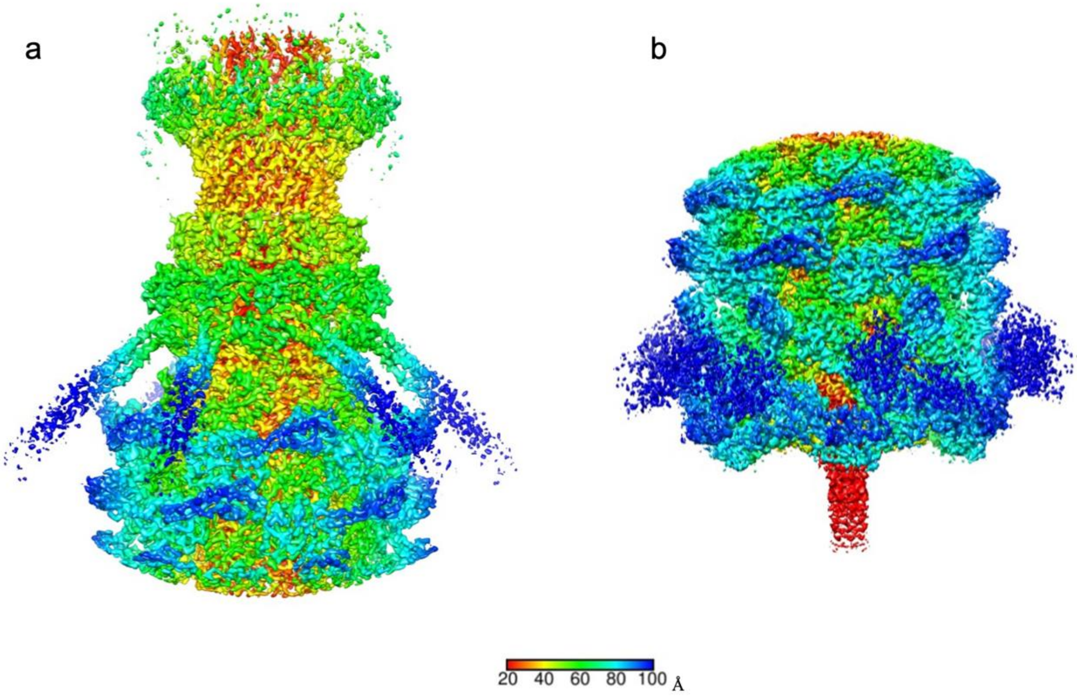

Supplementary Figure S1. Cryo-EM reconstructions of the XM1 neck region (a) and the baseplate region (b) calculated using 6-fold symmetry. The density is colored according to the distance from the 6-fold axis as indicated by the color bar.

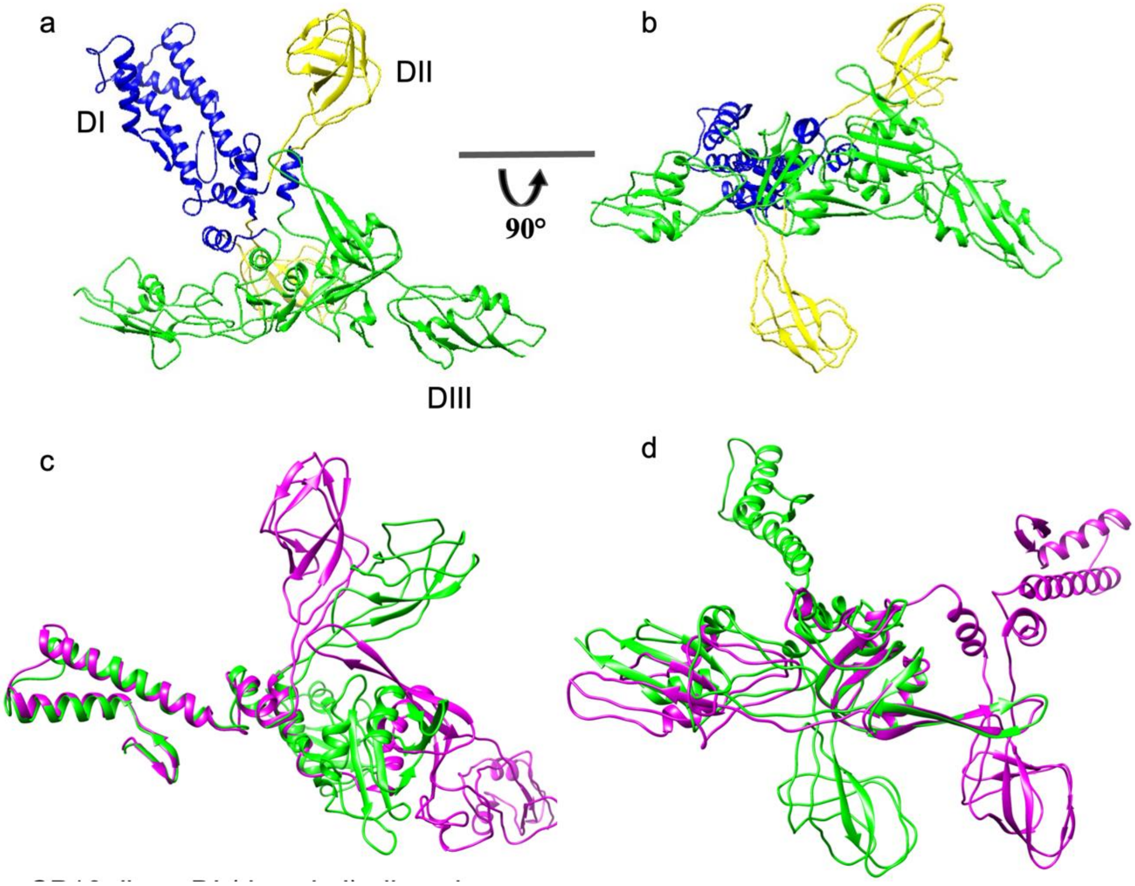

Supplementary Figure S2. Two views of the gp16 dimer. a and b: Domains I, II, and III are colored blue, yellow and green, respectively. The two copies of gp16 adopt different conformations. c and d: Superposition of the two copies of gp16 in the asymmetric unit, based on domain I (c) and domain III (d).

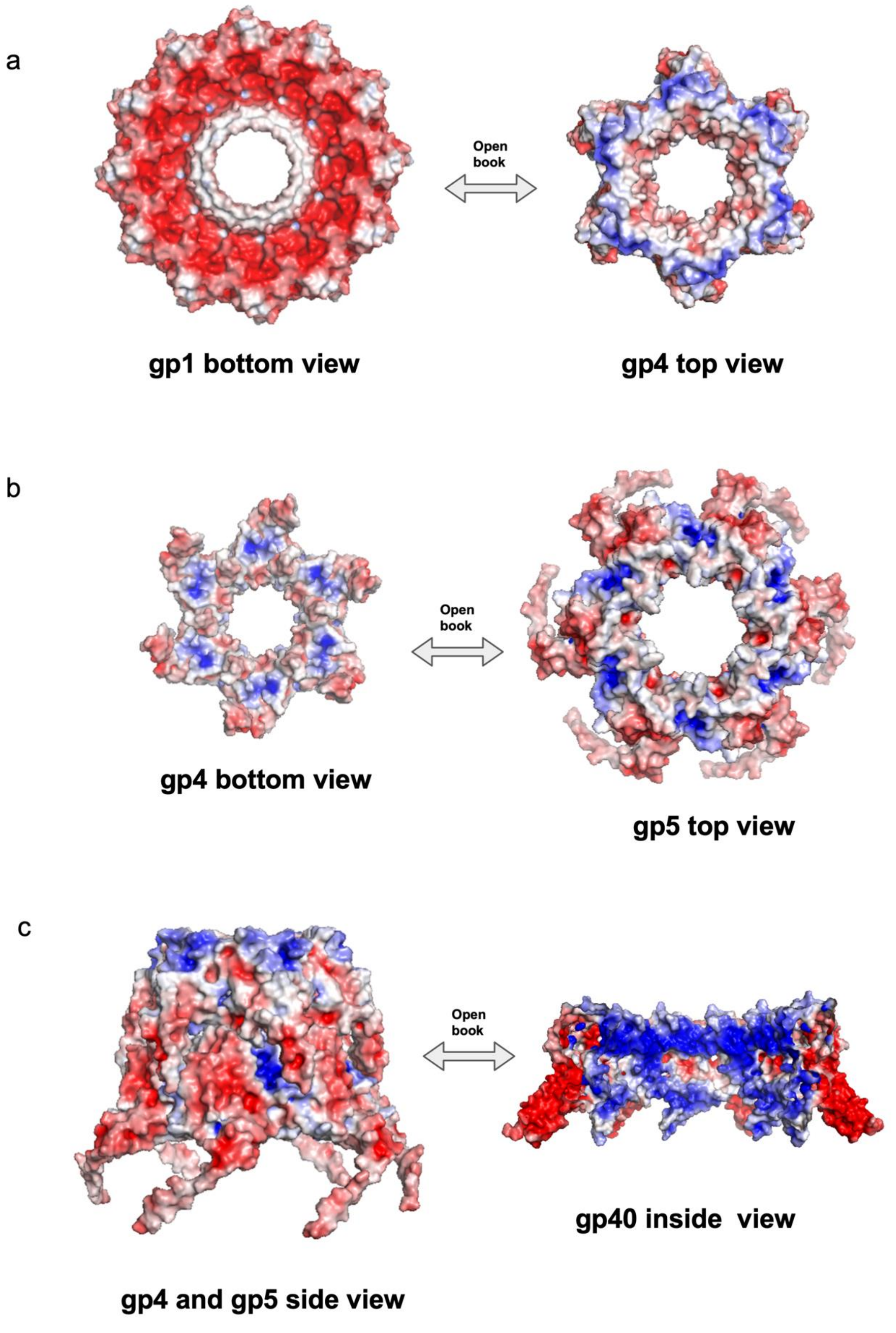
a
Open book
gp1 bottom view
gp4 top view
b
Open book
gp4 bottom view
gp5 top view
c
Open book
gp40 inside view
gp4 and gp5 side view
-5.000
5.000

Supplementary Figure S3. Electrostatic interactions between gp1, gp4, gp5, and gp40. a. The open book view of the molecular surfaces of gp1 and gp4 colored according to electrostatic potential. Red corresponds to a potential of -5 kT/e^- and blue corresponds to a potential of +5 kT/e^-. This shows that the bottom surface of gp1 is negatively charged and the top surface of gp4 is positively charged. Therefore, they tightly bind to each other. **b.** The open book view of the gp4 and gp5. The surfaces are colored according to electrostatic potential. The bottom surface of gp4 and the top surface of gp5 are both positively charged and do not show strong electrostatic attraction. **c.** The side surface of the gp4-gp5 complex (shown on the left) is negatively charged. The inner surface of the gp40 ring (shown on the right) is positively charged. Therefore, assembly of the gp40 ring around the gp4-gp5 complex reinforces the interactions between gp4 and gp5. As the

gp4 and gp5 proteins probably form the interface between the capsid and tail, gp40 is likely to reinforce the head-tail interactions.

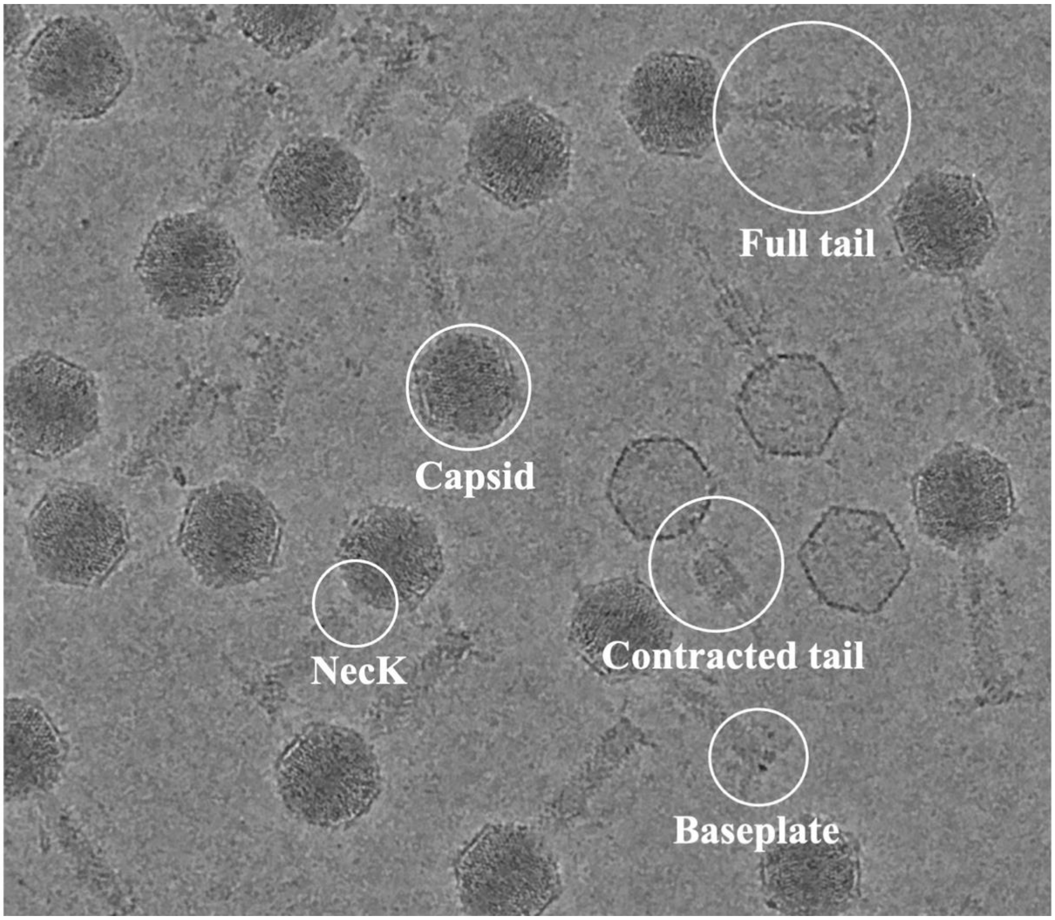

Supplementary Figure S4. Micrograph of the phage XM1 sample. White circles indicate particles boxed for reconstructions.

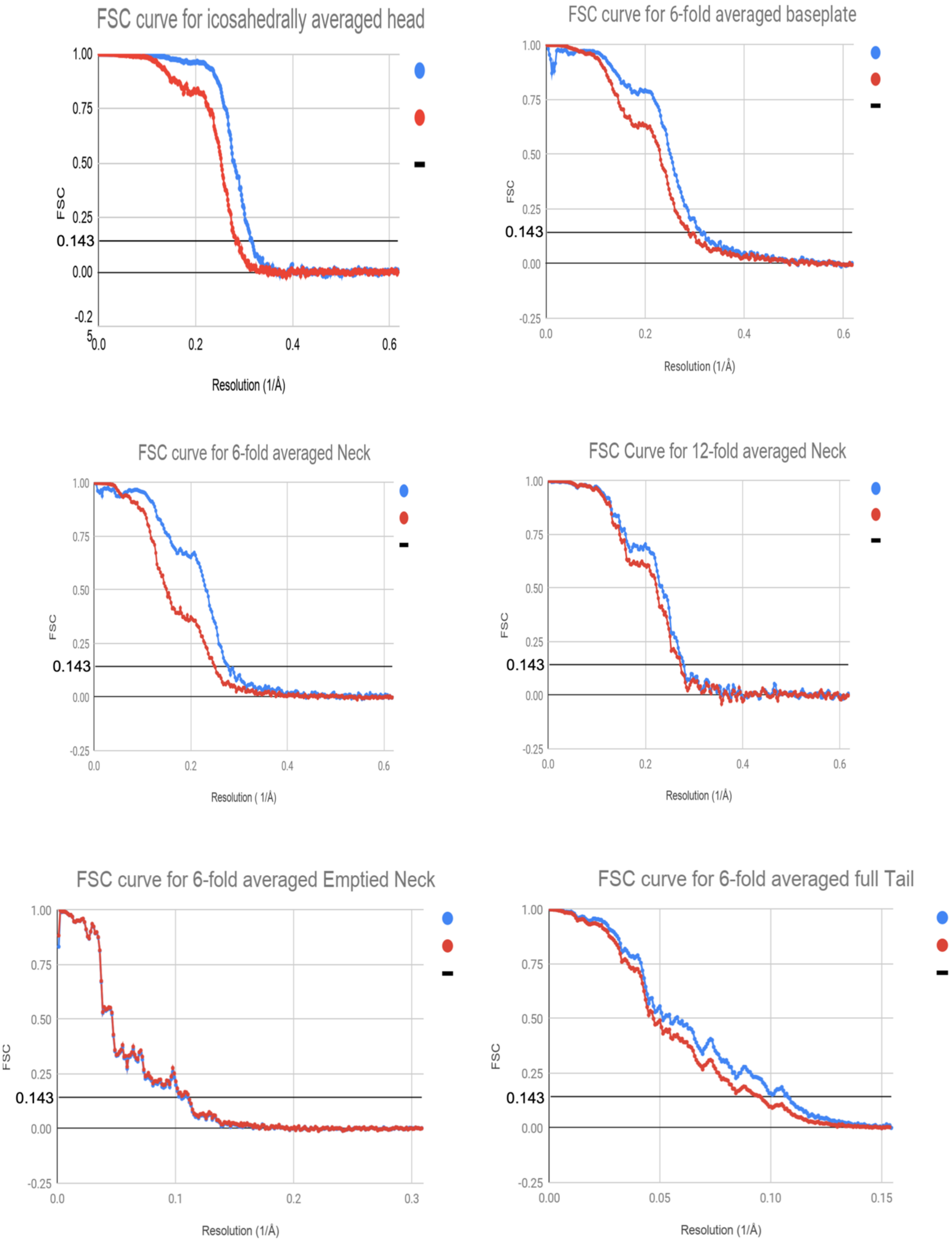

Supplementary Figure S5. Fourier shell correlation curves of the cryo-EM reconstructions.

Supplementary Table S1. Structural components of the XM1 virion.

	# of AA built/Total AA	Predicted function	HHPRED with probability score	Dali search with RMSD	phage T4 homologue	T6SS homologue
gp1	118AA/118AA	Head-tail Connector Protein	phage Spp1 gp15 (5A21_C), 91.41	phage Mu gp36 (5YDN), 2.9	gp13	-
gp4	114AA/114AA	Head-tail Connector Protein	phage Spp1 gp16 (5A21_E), 98.57	phage Spp1 gp16 (2KCA), 3.9	gp14	-
gp5	160AA/161AA	Tail terminator Protein	phage Spp1 gp17 (5A21_G), 97.05	phage Lambda gpU (3FZ2_E), 3.5	gp15	-
gp6	497AA/497AA	Tail Sheath Protein	R-type bacteriocin sheath protein (6GKW_A), 99.87	R-type pyocin sheath (3J9Q_A), 3.6	gp18	TssB/C, VipA/B
gp7	142AA/143AA	Tail Tube Protein	R-type bacteriocin tube protein (6GKX_A), 96.73	antifeeding prophage AFP1 (6RAP_A), 2.7	gp19	HCP
gp10	0/479AA	Tape Measure Protein	Phage 80a gp57 tape measure protein (6V8I_BF), 98.44		gp29	-
gp11	250AA/250AA	Baseplate Organizing Protein	Pyocin R2 tail tube protein(6U5B_K), 97.13	HCP1 (6BDC_A), 2.7	gp48/gp53	HCP
gp12	118AA/118AA	Baseplate Stabilizing Protein		Rift Valley Fever Virus Envelope protein (6EGT_A), 2.8	-	
gp13	0/376AA	Tail Hub Protein	Pyocin R2 tail hub protein (6U5H_A), 99.88		gp5/gp27	VgrG
gp14	0/240AA	Tail Puncturing Protein	phageSN gp41 puncturing protein (4RU3_A), 99.89		gp5.4	VgrG PAAR
gp15	110AA/117AA	Sheath initiator	phage T4 gp25 (5IW9_B), 99.75	phage T4 gp25 (5IW9_A), 5.0	gp25	TssE
gp16	404AA/404AA	Baseplate Wedge Protein	phage T4 gp7 (5HX2_D), 99.97	phage T4 gp6 (5HX2_D), 5.2	gp6	TssF
gp17	172AA/242AA (1-79, 107-200)	Baseplate Wedge Protein	Pyocin R2 tri1a (6U5B_w), 98.77	phage T4 gp7 (5HX2_A), 5.2	gp7	TssG

gp18	0/653AA	Tail Spike Protein	phage vb_AbaP_AS12 gp42 tailspike (6EU4_B), 95.52		-	-
gp40	126AA/839AA (1-126)	Collar Spike Protein	phage Det7 gp208 tailspike (6F7D_A), 99.71		-	-
gp49	365AA/412AA (9-337, 346-377)	Portal Protein	phage G20C portal protein (5NGD_D), 99.64	phage G20C portal protein (4ZJN_A), 4.0	gp20	-
gp53	160AA/160AA	Minor Capsid Protein		phage TW1 minor capsid protein (5WK1_K), 3.2	-	-
gp54	294AA/324AA (31-324)	Major Capsid Protein	phage TW1 major capsid protein (5WK1_C), 100	phage TW1 major capsid protein (5WK1_A), 3.8	gp23/gp24	-

Supplementary Table S2. Structure refinement statistics.

	EMDB map: 22931<br><br>PDB: 7KMX	EMDB map: 22917<br><br>PDB: 7KLN	EMDB map: 22896<br><br>PDB: 7KJK	EMDB map: 22873<br><br>PDB: 7KH1	EMDB map: 22960<br><br>--	EMDB map: 22959<br><br>--
**Data collection and processing**						
Voltage (kV)	300	300	300	300	300	300
Electron exposure ($e^-/Å^2$)	30	30	30	30	30	30
Defocus range (um)	0.5~2.5	0.5~2.5	0.5~2.5	0.5~2.5	0.5~2.5	0.5~2.5
Pixel size (Å)	0.82	0.82	0.82	0.82	3.24	3.24
Symmetry imposed	icosahedral	C12	C6	C6	C6	C6
Final particle images (no.)	19625	10615	10615	16068	1104	6844
Map resolution (Å) @FSC=0.143	3.2	3.6	3.6	3.2	9.1	10.6
**Refinement**						
Model composition					--	--
Non-hydrogen atoms	24045	45564	59088	97344	--	--
Protein residues	3178	5748	7740	12588	--	--
Ligands	0	0	0	0	--	--
R.M.S. deviations						
Bond lengths (Å)	0.006	0.007	0.009	0.009	--	--

Bond angles (º)	0.634	0.851	0.933	0.87	--	--
Validation						
MolProbity score	1.92	2.48	2.61	2.42	--	--
Clashscore	11.93	25.61	27.03	20.43	--	--
Poor rotamers (%)	0.12	0.48	1.45	0.35	--	--
Ramachandran						
Favored (%)	95.27	88.71	89.26	87.1	--	--
Allowed (%)	4.73	11.29	10.74	12.71	--	--
Disallowed (%)	0	0	0	0.19	--	--
